# Deletion of* Herpud1* Enhances Heme Oxygenase-1 Expression in a Mouse Model of Parkinson's Disease

**DOI:** 10.1155/2016/6163934

**Published:** 2016-02-23

**Authors:** Thuong Manh Le, Koji Hashida, Hieu Minh Ta, Mika Takarada-Iemata, Koichi Kokame, Yasuko Kitao, Osamu Hori

**Affiliations:** ^1^Department of Neuroanatomy, Kanazawa University Graduate School of Medical Science, Kanazawa, Ishikawa 920-8640, Japan; ^2^CREST, JST (Japan Science and Technology), Tokyo 102-8666, Japan; ^3^Department of Molecular Pathogenesis, National Cerebral and Cardiovascular Center, Osaka 565-8565, Japan

## Abstract

Herp is an endoplasmic reticulum- (ER-) resident membrane protein that plays a role in ER-associated degradation. We studied the expression of Herp and its effect on neurodegeneration in a mouse model of Parkinson's disease (PD), in which both the oxidative stress and the ER stress are evoked. Eight hours after administering a PD-related neurotoxin, 1-methyl-4-phenyl-1,2,3,6-tetrahydropyridine (MPTP), to mice, the expression of Herp increased at both the mRNA and the protein levels. Experiments using* Herpud1* ^+/+^ and* Herpud1* ^−/−^ mice revealed that the status of acute degeneration of nigrostriatal neurons and reactive astrogliosis was comparable between two genotypes after MPTP injection. However, the expression of a potent antioxidant, heme oxygenase-1 (HO-1), was detected to a higher degree in the astrocytes of* Herpud1* ^−/−^ mice than in the astrocytes of* Herpud1* ^+/+^ mice 24 h after MPTP administration. Further experiments using cultured astrocytes revealed that the stress response against MPP^+^, an active form of MPTP, and hydrogen peroxide, both of which cause oxidative stress, was comparable between the two genotypes. These results suggest that deletion of* Herpud1 *may cause a slightly higher level of initial damage in the nigrastrial neurons after MPTP administration but is compensated for by higher induction of antioxidants such as HO-1 in astrocytes.

## 1. Introduction

Parkinson's disease (PD) is a common neurodegenerative disease characterized pathologically by the loss of dopaminergic neurons and the presence of Lewy bodies (LB), or intraneuronal protein aggregates, in the substantia nigra pars compacta (SNpc) [1]. Recent studies utilizing genetic and environmental approaches have demonstrated that enhanced levels of intracellular stress such as oxidative stress, which is derived from mitochondrial dysfunction and/or dopamine metabolites, and endoplasmic reticulum (ER) stress, a condition where unfolded proteins accumulate in the ER [2], are involved in the pathogenesis of PD [3].

The mitochondrial toxin 1-methyl-4-phenyl-1,2,3,6-tetrahydropyridine (MPTP), which is widely used for creating an experimental PD model, causes both oxidative stress and ER stress, resulting in neurodegeneration in the SNpc [4–6]. Similarly, overexpression of *α*-synuclein, a familial PD-associated cytoplasmic protein, enhances the levels of both oxidative stress and ER stress [7] and affects the sensitivity of SNpc neurons to MPTP [8–10]. Overexpression of Parkin-associated endothelin receptor-like receptor (Pael-R) in the knockout mice of Parkin, another familial PD-associated protein, also causes both types of stress* in vitro *and* in vivo* [11, 12]. These lines of evidence suggest the existence of cross talk between the different intracellular stresses in the pathogenesis of PD, although the underlying mechanisms remain unclear.

Homocysteine-inducible, endoplasmic reticulum protein (Herp) is a membrane-bound, ubiquitin- (Ub-) like protein that is located in the endoplasmic reticulum (ER) of a variety of cells, including neurons [13–15].* Herpud1*, which encodes Herp, is strongly induced in response to ER stress [2] and is rapidly degraded by the proteasome [15]. Targeted disruption of the* Herpud1 *gene renders F9 embryonic carcinoma cells vulnerable to ER stress, suggesting that Herp plays a protective role against ER stress [15]. The function of Herp is not fully understood, but accumulating evidence suggests that it is involved in ER-associated degradation (ERAD), a system that clears unfolded or misfolded proteins in the ER via the ubiquitin-proteasome system and autophagy [2]. Several ERAD substrates were stabilized by the deletion of Herp [15–17] and Herp forms a complex with components of the ERAD machinery [18].

A study on PD reported that Herp was highly expressed both in neuronal and glial cells in the SNpc from patients with PD [19]. Gene silencing of Herp rendered PC12 cells vulnerable to 1-methyl-4-phenylpyridinium (MPP^+^), an active form of MPTP [20]. Furthermore, Herp counteracted mutant *α*-synuclein-induced ER stress via the homeostatic regulation of ER-resident calcium release channel proteins [21]. However, we recently reported that the deletion of Herp facilitated the degradation of *α*-synuclein and its binding partner synphilin-1 and improved cell viability during proteasomal inhibition [22]. Another recent report also demonstrated that Herp depletion protected cells from protein aggregation by upregulating autophagy [23]. To understand the details of the role Herp plays in PD, we analyzed the expression and possible involvement of Herp in an experimental model of PD using wild-type (*Herpud1* ^+/+^) and* Herpud1* ^−/−^ mice.

## 2. Materials and Methods

### 2.1. Mice and the MPTP Injection PD Model

All animal care and handling procedures were approved by the Animal Care and Use Committee of Kanazawa University.* Herpud1* ^−/−^ micewere generated as described previously [24] and backcrossed to the C57BL/6 strain over eight times.* Herpud1* ^+/+^ and* Herpud1* ^−/−^ male mice (aged 10–14 weeks and weighing 25–30 g) were used for the experiments. The acute MPTP injection PD model was created by administering intraperitoneal injections of MPTP (20 mg/kg) four times at 2 h intervals, as described previously [25]. At 0, 8, 24, and 72 h after MPTP injection, brain samples were prepared for the quantitative real-time reverse transcription polymerase chain reaction (qRT-PCR), western blotting, and histological analyses.

### 2.2. Cell Culture

Astrocytes were isolated from the cerebral cortex of 1- to 3-day-old neonatal mice following a previously described method with minor modifications [26]. Briefly, the cerebral hemispheres were harvested from neonatal mice, and the meninges were carefully removed. Then, the brain tissue was digested at 37°C in HEPES-buffered saline containing Dispase II (2 mg/mL; Wako, Osaka, Japan). Cells were collected by centrifugation and resuspended in minimum essential medium (Sigma, St. Louis, MO, USA) supplemented with 10% fetal bovine serum. After a 10-day cultivation period, microglial cells were removed by aspiration after shaking and the adherent cell population was collected and used for experiments. When the cultures achieved confluence, cells were treated with MPP^+^ (Sigma) or hydrogen peroxide (Nacalai Tesque Inc., Kyoto, Japan) for 16 h, both of which cause oxidative stress. The cells were collected and subjected to western blotting.

### 2.3. qRT-PCR

Total RNA was extracted from the ventral midbrain or caudate putamen (CPu) of each mouse using the RNeasy Lipid Tissue Mini Kit (Qiagen, Valencia, CA, USA) or from cultured astrocytes using the RNeasy Mini Kit (Qiagen). RT reactions containing 1 *μ*g of total RNA were performed using PrimeScript (Takara, Shiga, Japan). cDNA was amplified with THUNDERBIRD*™* SYBR qPCR® Mix (TOYOBO Co., Ltd., Osaka, Japan) by using specific primers for* Herpud1*,* Hmox1*,* Nfe2l2*,* Hspa5*, and* Actb*. The comparative Ct method was used to analyze the data with MxPro 4.10 (Agilent Technologies, Santa Clara, CA, USA). The values for each gene were normalized to the* Actb* expression levels. The sequences of the primers that were used for qRT-PCR are listed in Supplemental Table 1 (in Supplementary Material available online at http://dx.doi.org/10.1155/2016/6163934).

### 2.4. Western Blotting

Samples from the CPu or from cultured astrocytes were solubilized in buffer containing 1% NP40, 0.1% sodium dodecyl sulfate, and 0.2% deoxycholate and were subjected to western blotting with the following antibodies: tyrosine hydroxylase (TH; EMD Millipore, Billerica, MA, USA), glial fibrillary acidic protein (GFAP; Dako, Glostrup, Denmark), GRP78 (StressGen, Victoria, British Columbia, Canada), heme oxygenase-1 (HO-1; Abcam, Cambridge, UK), and *β*-actin (Sigma). Primary antibody binding was visualized using the ECL system (GE Healthcare Bio-Sciences Corp., Piscataway, NJ, USA).

### 2.5. Immunohistochemistry

Immunohistochemical analysis was performed as previously described [27]. In brief, brains were removed from mice after perfusion with 4% paraformaldehyde and postfixed in the same fixative for 4 hours at 4°C. After being cryoprotected in 30% sucrose, brains were cut in serial coronal 10 *μ*m-thick sections containing the CPu (from Bregma +1.34 mm to Bregma +0.26 mm) and the midbrain covering the whole SNpc (from Bregma −2.80 mm to Bregma −3.80 mm) on a cryostat. Brain sections were mounted in series on ten slides (around ten sections were mounted on each slide). One out of these ten slides, representing a set of sections 100 *μ*m apart, was processed for immunohistochemistry with the following antibodies: TH, GFAP, GRP78, HO-1, Ub (StressGen), *α*-synuclein (BD, Franklin Lakes, NJ, USA), and LC3B (Cell Signaling Technology, Danvers, MA, USA). In some cases, the cell nuclei were visualized with 4′,6-diamidino-2-phenylindole (DAPI; Sigma). Alexa488-conjugated (Thermo Fisher Scientific, Rockford, IL, USA) or Cy3-conjugated (Jackson ImmunoResearch Laboratories, West Grove, PA, USA) secondary antibody was used for visualization of immunolabeling.

### 2.6. Image Quantification

Quantification of the western blots and immunohistochemical analyses was performed using Image J (version 1.42, Wayne Rasband, National Institutes of Health, Bethesda, Maryland, USA). TH-positive neurons were identified by colocalization of TH with DAPI, and the number in the SNpc was counted in four representative sections out of ten sections mounted on one slide, which covered the whole SNpc.

### 2.7. Statistical Analysis

Statistical analyses were performed using Bonferroni/Dunn tests following a one-way analysis of variance.

## 3. Results

### 3.1. Expression of Herp after MPTP Administration

**Figure 1:**
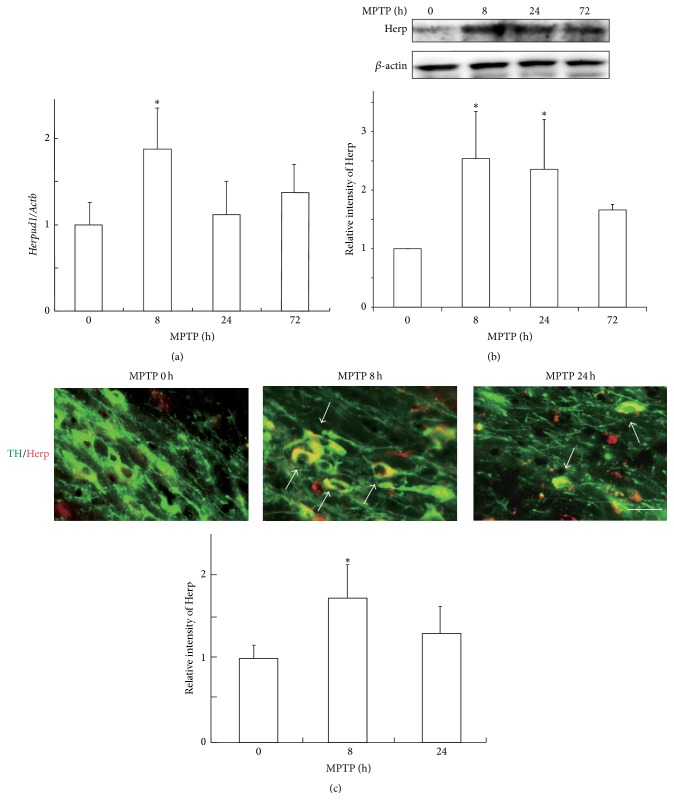
Expression of Herp after MPTP administration. (a) qRT-PCR.* Herpud1* ^+/+^ mice were injected with MPTP, and the total RNA (1 *μ*g), which was extracted from the ventral midbrain, was subjected to qRT-PCR. Each value shown is the mean ± the standard deviation (SD) (*n* = 4), and the value derived from the mice without MPTP injection is designated as one. ^*∗*^ *P* < 0.05, compared to mice without MPTP administration. (b) Western blots.* Herpud1* ^+/+^ mice were injected with MPTP, and protein samples (30 *μ*g) extracted from the CPu were subjected to western blotting with anti-Herp and anti-*β*-actin antibodies. Each value shown is the mean ± SD (*n* = 4), and the value derived from the mice without MPTP administration is designated as one. ^*∗*^ *P* < 0.05, compared to mice without MPTP administration. (c) Immunohistochemistry.* Herpud1* ^+/+^ mice were injected with MPTP and perfused with 4% paraformaldehyde at the indicated times. Brain sections, including sections with the SNpc, were analyzed by immunohistochemistry with anti-Herp and anti-TH antibodies. Arrows indicate Herp expression in the TH-positive dopaminergic neurons after MPTP administration. Scale bars = 30 *μ*m.

Eight hours after mice were intraperitoneally injected with MPTP, the expression of Herp in the nigrostriatal system increased at both the mRNA and protein levels (Figures 1(a) and 1(b)). Immunohistochemical analysis revealed that the expression of Herp was enhanced mainly in the TH-positive neurons in the SNpc after MPTP injection (Figure 1(c)). These results suggest that Herp may function at the relatively early phases in the nigrostriatal neurons after MPTP injection.

### 3.2. Effects of* Herpud1* Deletion on the Neurodegeneration and Astroglial Activation after MPTP Administration

**Figure 2:**
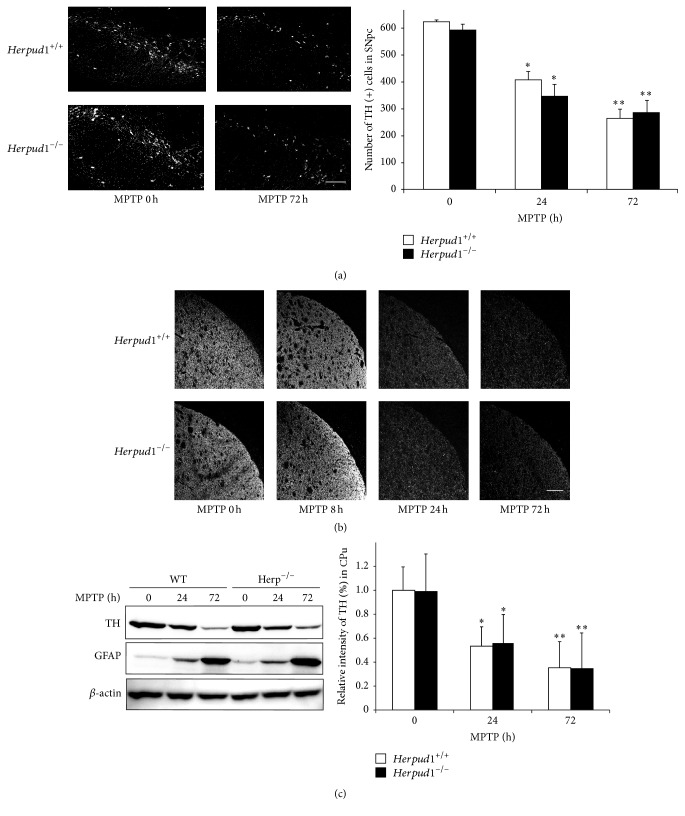
Neurodegeneration after MPTP administration. (a, b) Immunohistochemistry.* Herpud1* ^+/+^ and* Herpud1* ^−/−^ mice were injected with MPTP and perfused with 4% paraformaldehyde at the indicated times. Brain sections, including sections with the SNpc (a) or CPu (b), were subjected to immunohistochemistry with anti-TH antibody. The number of TH-positive neurons in the SNpc is shown in (a). Values shown are the mean ± SD (*n* = 4). ^*∗∗*^ *P* < 0.01, compared to mice without MPTP administration. (c) Western blots.* Herpud1* ^+/+^ and* Herpud1* ^−/−^ mice were injected with MPTP, and protein samples (30 *μ*g) extracted from the CPu were subjected to western blotting with anti-TH, anti-GFAP, and anti-*β*-actin antibodies. The relative intensity of TH is shown in the graph. Each value shown is the mean ± SD (*n* = 4), and the value derived from* Herpud1* ^+/+^ mice without MPTP injection is designated as one. ^*∗*^ *P* < 0.05, compared to mice without MPTP administration. Scale bars = 100 *μ*m.

To evaluate the role of Herp in MPTP-induced neurodegeneration and astroglial activation,* Herpud1* ^+/+^ and* Herpud1* ^−/−^ mice were intraperitoneally injected with MPTP. Immunohistochemical analysis revealed that the number of TH-positive cells in the SNpc decreased to a similar level between the two genotypes (Figure 2(a)). Approximately 50–60% and 40–50% of TH-positive cells were observed at 24 and 72 h after MPTP administration, respectively, in both genotypes (Figure 2(a)). Moreover, the expression of TH in the CPu decreased in a similar manner between the two genotypes after MPTP injection (Figure 2(b)). Western blot analysis confirmed the reduction of TH expression in both genotypes after MPTP administration (Figure 2(c)). Since it is well known that astrocytes are activated in response to MPTP administration, we next analyzed the status of astrocytes using western blots (Figure 2(c)) and immunohistochemistry (Figure S1A). Both experiments revealed that the expression of GFAP, a marker of astroglial activation, increased in a similar manner in both genotypes after MPTP administration. These results suggest that the levels of acute nigrostriatal dopaminergic neuron degeneration and reactive astrogliosis were comparable between the two genotypes after MPTP injection.

### 3.3. Effects of* Herpud1* Deletion on the Stress Response and Protein Degradation after MPTP Administration

**Figure 3:**
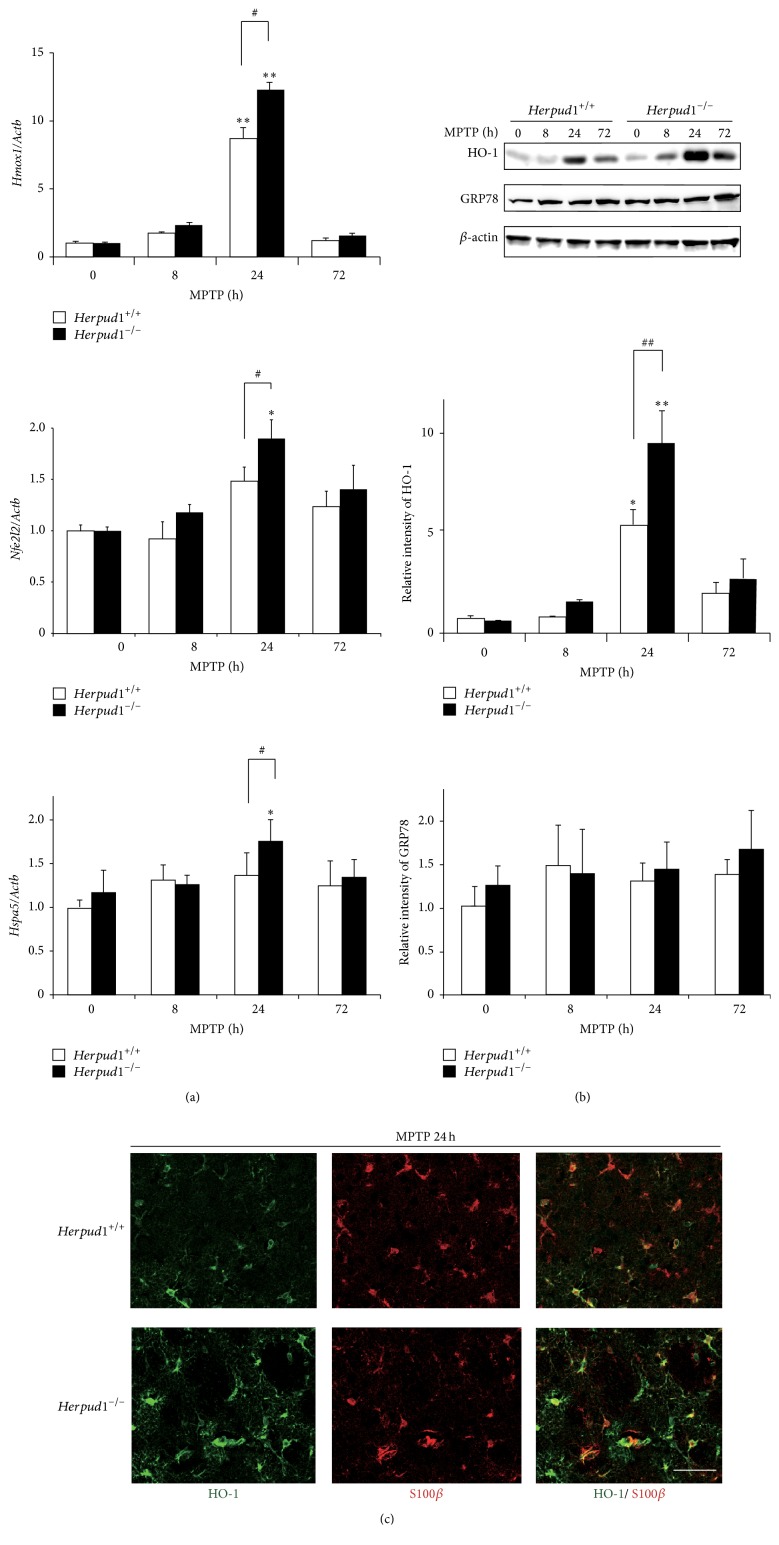
Expression of stress-related genes after MPTP administration. (a) qRT-PCR. (b) Western blots.* Herpud1* ^+/+^ and* Herpud1* ^−/−^ mice were injected with MPTP, and the total RNA (1 *μ*g) or protein samples (30 *μ*g) extracted from the CPu were subjected to qRT-PCR (a) or western blotting (*n* = 4) (b). Each value shown is the mean ± SD, and the value derived from* Herpud1* ^+/+^ mice without MPTP administration is designated as one. ^*∗*^ *P* < 0.05; ^*∗∗*^ *P* < 0.01, compared to mice without MPTP administration. ^#^ *P* < 0.05; ^##^ *P* < 0.01 between the two genotypes. (c) Immunohistochemistry.* Herpud1* ^+/+^ and* Herpud1* ^−/−^ mice were injected with MPTP and perfused with 4% paraformaldehyde after 24 h. Brain sections were subjected to immunohistochemistry with anti-HO-1 and anti-S100*β* antibodies. Scale bars = 50 *μ*m (c).

As* Herpud1 *is a stress-responsive gene, the status of the intracellular stress response in the CPu was compared between the two genotypes. Although the deletion of* Herpud1* did not affect MPTP-induced neurodegeneration, qRT-PCR revealed that the expression of oxidative stress-related genes such as* Hmox1* and* Nfe2l2*, which encode HO-1 and Nrf2, respectively, was highly induced in* Herpud* ^−/−^ mice compared to that in* Herpud1* ^+/+^ mice 24 h after MPTP administration (Figure 3(a)). Similarly, expression of the ER stress-related gene* Hspa5*, which encodes a molecular chaperone in the ER GRP78, was higher in* Herpud1* ^−/−^ mice compared to that in* Herpud1* ^+/+^ mice 24 h after MPTP administration (Figure 3(a)). Western blot analysis confirmed the higher level of HO-1 expression, but not of GRP78 expression, in* Herpud1* ^−/−^ mice 24 h after MPTP administration (Figure 3(b)). Immunohistochemical analyses revealed that, in the CPu, HO-1 was predominantly located in the astrocytes in both genotypes after MPTP administration (Figure 3(c)). In contrast, in the SNpc, HO-1 was located mainly in neurons in* Herpud1* ^+/+^ mice and in both neurons and astrocyte-like cells in* Herpud1* ^−/−^ mice after MPTP administration (Figure S1B). These results suggest that the deletion of* Herpud1* enhanced the oxidative stress response in astrocytes after MPTP administration.

As Herp likely functions in the ERAD, and since deletion of the* Herpud1* gene facilitated the degradation of *α*-synuclein in some type of cells [22], the status of Ub, *α*-synuclein, and LC3 was compared between the two genotypes. Western blot analysis failed to detect the accumulation of Ub/ubiquitinated proteins (Figure S2A) or of *α*-synuclein (Figure S2B) in either genotype after MPTP administration. Similarly, activation of LC3 was not observed in either genotype after MPTP administration (Figure S2C).

### 3.4. Effects of* Herpud1* Deletion on the Cultured Astrocytes

**Figure 4:**
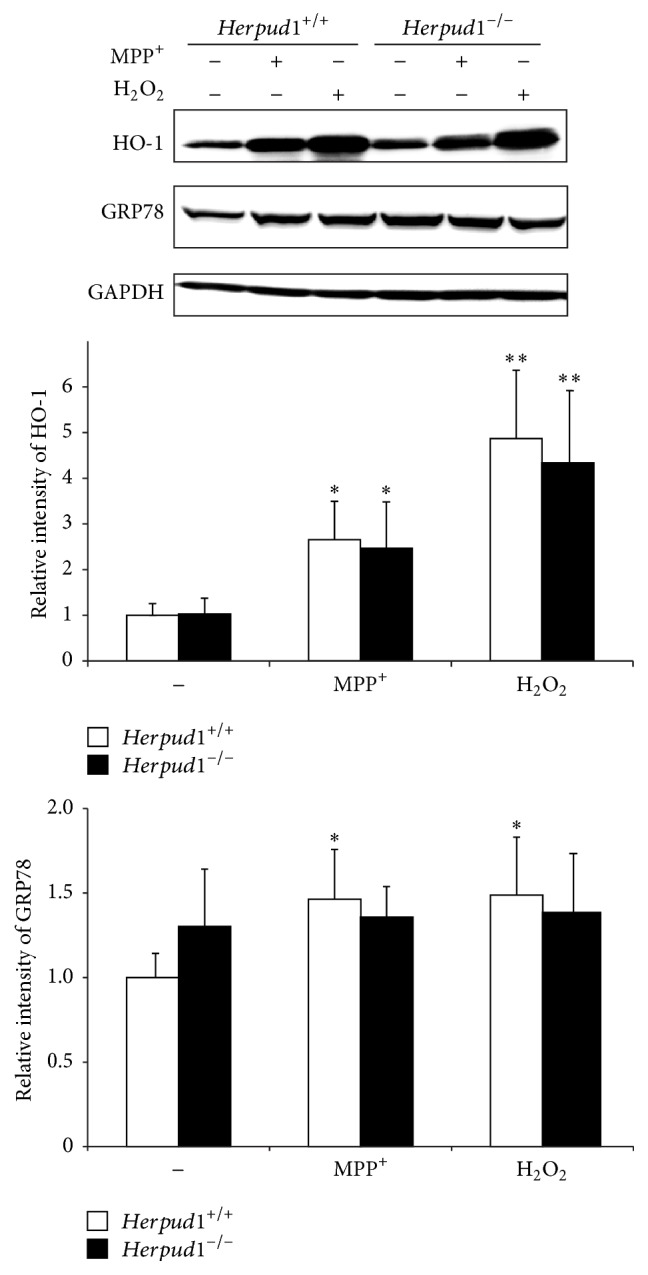
Expression of stress-related genes in cultured astrocytes.* Herpud1* ^+/+^ and* Herpud1* ^−/−^ mice-derived astrocytes were treated with MPP^+^ for 16 h, and, after cell lysis, protein samples (30 *μ*g/sample) were subjected to western blotting for HO-1, GRP78, and GAPDH. Each value shown is the mean ± SD (*n* = 4), and the value derived from* Herpud1* ^+/+^ astrocytes without MPP^+^ treatment is designated as one. ^*∗*^ *P* < 0.05; ^*∗∗*^ *P* < 0.01, compared to cells without MPP^+^ treatment.

To clarify whether the phenotypes of* Herpud1* ^−/−^ mice were associated with the enhanced level of initial brain damage or with the altered astroglial stress response, we employed cultured astrocytes derived from neonatal mouse brains. When astrocytes were challenged to MPP^+^, an active form of MPTP, or hydrogen peroxide, which causes oxidative stress, the expression of HO-1 increased to similar levels in both genotypes (Figure 4). The expression of GRP78 also mildly increased in* Herpud1* ^+/+^ cells but not in* Herpud1* ^−/−^ cells in the same conditions. These results suggest that the phenotype observed in* Herpud1* ^−/−^ mice may be associated with a slightly higher level of initial damage in the nigrostriatal neurons after MPTP administration rather than associated with the altered stress response against oxidative stress in the* Herpud1* ^−/−^ astrocytes.

## 4. Discussion

In this study, we demonstrated that the expression of Herp increased 8 h after MPTP administration at both the mRNA and protein levels. Experiments using* Herpud1* ^+/+^ and* Herpud* ^−/−^ mice revealed that the levels of acute nigrostriatal dopaminergic neuron degeneration and reactive astrogliosis were comparable between the two genotypes after MPTP injection. However, expression of the potent antioxidant gene* Hmox1 *was detected to a higher degree in the astrocytes of* Herpud1* ^−/−^ mice than in the astrocytes of* Herpud1* ^+/+^ mice 24 h after MPTP administration. Further experiments using cultured astrocytes revealed that the stress response against MPP^+^ and hydrogen peroxide was comparable between the two genotypes. These results suggest that deletion of* Herpud1 *may cause a slightly higher level of initial damage or oxidative stress in nigrostriatal neurons after MPTP administration but that this is compensated for by the higher induction of antioxidative genes including* Hmox1* in astrocytes.

Our collaborators and we previously reported that ORP150, a molecular chaperone in the ER, and ATF6*α*, a key transcriptional factor in the unfolded protein response, are important for the survival of and protein degradation in nigrostriatal dopaminergic neurons and for the subsequent reactive astrogliosis after MPTP administration [12, 27–29]. Therefore, we started our study by analyzing in which cells and at what time points Herp functions after MPTP administration. Our results suggest that Herp may function in the nigrostriatal neurons at relatively early periods after MPTP administration (Figure 1). After converting to its active form, MPP^+^, MPTP causes oxidative stress in the nigrostriatal neurons as an early event [6], which, in turn, causes accumulation of the unfolded proteins in the ER, either by producing oxidative protein modifications or by altering the redox status in the ER [4, 5, 30]. In this study, the upregulation of* Herpud1 *was stronger than that of* Hspa5* after MPTP administration (Figures 1(a)–1(c) and 3(a), 3(b)), although they are both unfolded protein response target genes. This may indicate the existence of a Herp-specific role in the nigrostriatal neurons after MPTP administration. Although the precise mechanism for the* Herpud1* upregulation is not clear, one possibility is that ERSE-II [31], an ER stress-responsive cis-element found in the* Herpud1* promoter, but not in the* Hepa5 *promoter, may play an important role after MPTP administration.

Several reports have demonstrated the neuroprotective role of Herp in pathological conditions such as brain ischemia and PD [20, 21, 24]. However, our results suggest that Herp is not essential for the survival of nigrostriatal neurons or glial activation after MPTP administration. One possible explanation for this discrepancy is that we employed* Herpud1* ^−/−^ mice as a tool for analyzing the role of Herp after MPTP administration, wherein long-term gene deletion may cause some compensatory responses such as higher levels of HO-1 induction after MPTP administration, thus masking the phenotypes that are supposed to be observed.

HO-1 is an enzyme that degrades heme to biliverdin, free iron, and carbon monoxide (CO). In PD, HO-1 is highly expressed in astrocytes in the SNpc and exerts both beneficial and toxic functions [32, 33]. It is known that HO-1 protects neurons by producing antioxidant biliverdin, or by enhancing production of neurotrophic factors such as BDNF and GDNF [34]. However, recent evidence also demonstrated that hyperactivation of HO-1 in astrocytes leads to mitochondrial sequestration of iron and may contribute to the pathological iron deposition and bioenergy failure [33]. Therefore, one of our future studies will be to compare the phenotypes of* Herpud1* ^+/+^ and* Herpud1* ^−/−^ mice in the chronic phase after MPTP administration. It is also intriguing to study the effect of HO-1 inhibitors such as imidazole-dioxolane on* Herpud1* ^−/−^ mice [35].

In this study, deletion of* Herpud1* did not cause Ub-positive or *α*-synuclein-positive protein aggregation or alterations (Figure S2). This is in contrast with other recent findings showing that* ATF6α* deletion led to the accumulation of Ub-positive protein aggregates in nigrostriatal neurons after MPTP administration [27, 29]. These results suggest that other AFT6*α* target genes may be required to link ERAD to Ub-positive protein aggregation.

In conclusion, we found upregulation of* Herpud1 *in the nigrostriatal neurons at relatively early phases after MPTP administration. Deletion of* Herpud1* may induce a slightly higher level of initial damage or oxidative stress in the nigrostriatal neurons after MPTP administration, but this is compensated for by a higher induction of antioxidative genes including* Hmox1* in astrocytes.

## Supplementary Material

Supplemental materials include supplemental figure legends, supplemental figures (Fig. S1 and Fig. S2) and supplemental table 1 which is the list of PCR primers.

## References

[1] Dauer W., Przedborski S. (2003). Parkinson's disease: mechanisms and models. *Neuron*.

[2] Schröder M., Kaufman R. J. (2005). The mammalian unfolded protein response. *Annual Review of Biochemistry*.

[3] Thomas B., Beal M. F. (2007). Parkinson's disease. *Human Molecular Genetics*.

[4] Ryu E. J., Harding H. P., Angelastro J. M., Vitolo O. V., Ron D., Greene L. A. (2002). Endoplasmic reticulum stress and the unfolded protein response in cellular models of Parkinson's disease. *The Journal of Neuroscience*.

[5] Holtz W. A., Turetzky J. M., Jong Y.-J. I., O'Malley K. L. (2006). Oxidative stress-triggered unfolded protein response is upstream of intrinsic cell death evoked by parkinsonian mimetics. *Journal of Neurochemistry*.

[6] Zhou C., Huang Y., Przedborski S. (2008). Oxidative stress in Parkinson's disease: a mechanism of pathogenic and therapeutic significance. *Annals of the New York Academy of Sciences*.

[7] Smith W. W., Jiang H., Pei Z. (2005). Endoplasmic reticulum stress and mitochondrial cell death pathways mediate A53T mutant alpha-synuclein-induced toxicity. *Human Molecular Genetics*.

[8] Song D. D., Shults C. W., Sisk A., Rockenstein E., Masliah E. (2004). Enhanced substantia nigra mitochondrial pathology in human *α*-synuclein transgenic mice after treatment with MPTP. *Experimental Neurology*.

[9] Klivenyi P., Siwek D., Gardian G. (2006). Mice lacking alpha-synuclein are resistant to mitochondrial toxins. *Neurobiology of Disease*.

[10] Nieto M., Gil-Bea F. J., Dalfó E. (2006). Increased sensitivity to MPTP in human *α*-synuclein A30P transgenic mice. *Neurobiology of Aging*.

[11] Imai Y., Soda M., Inoue H., Hattori N., Mizuno Y., Takahashi R. (2001). An unfolded putative transmembrane polypeptide, which can lead to endoplasmic reticulum stress, is a substrate of Parkin. *Cell*.

[12] Kitao Y., Imai Y., Ozawa K. (2007). Pael receptor induces death of dopaminergic neurons in the substantia nigra via endoplasmic reticulum stress and dopamine toxicity, which is enhanced under condition of parkin inactivation. *Human Molecular Genetics*.

[13] Kokame K., Agarwal K. L., Kato H., Miyata T. (2000). Herp, a new ubiquitin-like membrane protein induced by endoplasmic reticulum stress. *The Journal of Biological Chemistry*.

[14] Sai X., Kawamur Y., Kokame K. (2002). Endoplasmic reticulum stress-inducible protein, Herp, enhances presenilin-mediated generation of amyloid *β*-protein. *The Journal of Biological Chemistry*.

[15] Hori O., Ichinoda F., Yamaguchi A. (2004). Role of Herp in the endoplasmic reticulum stress response. *Genes to Cells*.

[16] Okuda-Shimizu Y., Hendershot L. M. (2007). Characterization of an ERAD pathway for nonglycosylated BiP substrates, which require Herp. *Molecular Cell*.

[17] Kim T.-Y., Kim E., Yoon S. K., Yoon J.-B. (2008). Herp enhances ER-associated protein degradation by recruiting ubiquilins. *Biochemical and Biophysical Research Communications*.

[18] Schulze A., Standera S., Buerger E. (2005). The ubiquitin-domain protein HERP forms a complex with components of the endoplasmic reticulum associated degradation pathway. *Journal of Molecular Biology*.

[19] Slodzinski H., Moran L. B., Michael G. J. (2009). Homocysteine-induced endoplasmic reticulum protein (Herp) is up-regulated in parkinsonian substantia nigra and present in the core of Lewy bodies. *Clinical Neuropathology*.

[20] Chigurupati S., Wei Z., Belal C. (2009). The homocysteine-inducible endoplasmic reticulum stress protein counteracts calcium store depletion and induction of CCAAT enhancer-binding protein homologous protein in a neurotoxin model of Parkinson Disease. *The Journal of Biological Chemistry*.

[21] Belal C., Ameli N. J., El kommos A. (2012). The homocysteine-inducible endoplasmic reticulum (ER) stress protein herp counteracts mutant *α*-synuclein-induced ER stress via the homeostatic regulation of ER-resident calcium release channel proteins. *Human Molecular Genetics*.

[22] Miura H., Hashida K., Sudo H. (2010). Deletion of Herp facilitates degradation of cytosolic proteins. *Genes to Cells*.

[23] Quiroga C., Gatica D., Paredes F. (2013). Herp depletion protects from protein aggregation by up-regulating autophagy. *Biochimica et Biophysica Acta—Molecular Cell Research*.

[24] Eura Y., Yanamoto H., Arai Y., Okuda T., Miyata T., Kokame K. (2012). Derlin-1 deficiency is embryonic lethal, derlin-3 deficiency appears normal, and herp deficiency is intolerant to glucose load and ischemia in mice. *PLoS ONE*.

[25] Kühn K., Wellen J., Link N., Maskri L., Lübbert H., Stichel C. C. (2003). The mouse MPTP model: gene expression changes in dopaminergic neurons. *The European Journal of Neuroscience*.

[26] Hori O., Matsumoto M., Maeda Y. (1994). Metabolic and biosynthetic alterations in cultured astrocytes exposed to hypoxia/reoxygenation. *Journal of Neurochemistry*.

[27] Hashida K., Kitao Y., Sudo H. (2012). ATF6alpha promotes astroglial activation and neuronal survival in a chronic mouse model of Parkinson's disease. *PLoS ONE*.

[28] Kitao Y., Hashimoto K., Matsuyama T. (2004). ORP150/HSP12A regulates Purkinje cell survival: a role for endoplasmic reticulum stress in cerebellar development. *The Journal of Neuroscience*.

[29] Egawa N., Yamamoto K., Inoue H. (2011). The endoplasmic reticulum stress sensor, ATF6*α*, protects against neurotoxin-induced dopaminergic neuronal death. *The Journal of Biological Chemistry*.

[30] Holtz W. A., O'Malley K. L. (2003). Parkinsonian mimetics induce aspects of unfolded protein response in death of dopaminergic neurons. *The Journal of Biological Chemistry*.

[31] Kokame K., Kato H., Miyata T. (2001). Identification of ERSE-II, a new cis-acting element responsible for the ATF6-dependent mammalian unfolded protein response. *The Journal of Biological Chemistry*.

[32] Schipper H. M. (2000). Heme oxygenase-1: role in brain aging and neurodegeneration. *Experimental Gerontology*.

[33] Schipper H. M., Song W., Zukor H., Hascalovici J. R., Zeligman D. (2009). Heme oxygenase-1 and neurodegeneration: expanding frontiers of engagement. *Journal of Neurochemistry*.

[34] Song W., Zukor H., Lin S.-H. (2012). Unregulated brain iron deposition in transgenic mice over-expressing HMOX1 in the astrocytic compartment. *Journal of Neurochemistry*.

[35] Csongradi E., Vera T., Rimoldi J. M., Gadepalli R. S. V., Stec D. E. (2010). In vivo inhibition of renal heme oxygenase with an imidazole-dioxolane inhibitor. *Pharmacological Research*.

