# Supplementary material for: Deletion of Herpud1 Enhances Heme Oxygenase-1 Expression in a Mouse Model of Parkinson's Disease
Source: Parkinsons Dis. 2016 Feb 23;2016:6163934. doi: 10.1155/2016/6163934 (PMC4781986; doi:10.1155/2016/6163934)
Supplement: Supplementary file 1 — Supplemental materials include supplemental figure legends, supplemental figures (Fig. S1 and Fig. S2) and supplemental table 1 which is the list of PCR primers. [file 6163934.f1.pdf]

Deletion of *Herpud1* enhances heme oxygenase-1 expression in a mouse model of Parkinson's disease

Supplementary FIGURE LEGENDS

Figure S1. Astroglial activation in the CPu and HO-1 expression in the SNpc. *Herpud1*$^{+/+}$ and *Herpud1* $^{-/-}$ mice were injected with MPTP and perfused with 4% paraformaldehyde at the indicated times. Brain sections, including sections with the CPu (A) or SNpc (B), were subjected to immunohistochemistry with the indicated antibodies. Arrows and arrowheads indicate HO-1 expression in the TH-positive dopaminergic neurons and astrocyte-like cells after MPTP administration, respectively. Scale bars = 150μm (A), 30μm (B).

Figure S2. Gene expression associated with protein degradation after MPTP administration. *Herpud1*$^{+/+}$ and *Herpud1* $^{-/-}$ mice were injected with MPTP and protein samples (30 μg) extracted from the CPu were subjected to western blotting with anti- Ub (A), anti-α-synuclein (B) and anti-LC3 (C) antibodies.

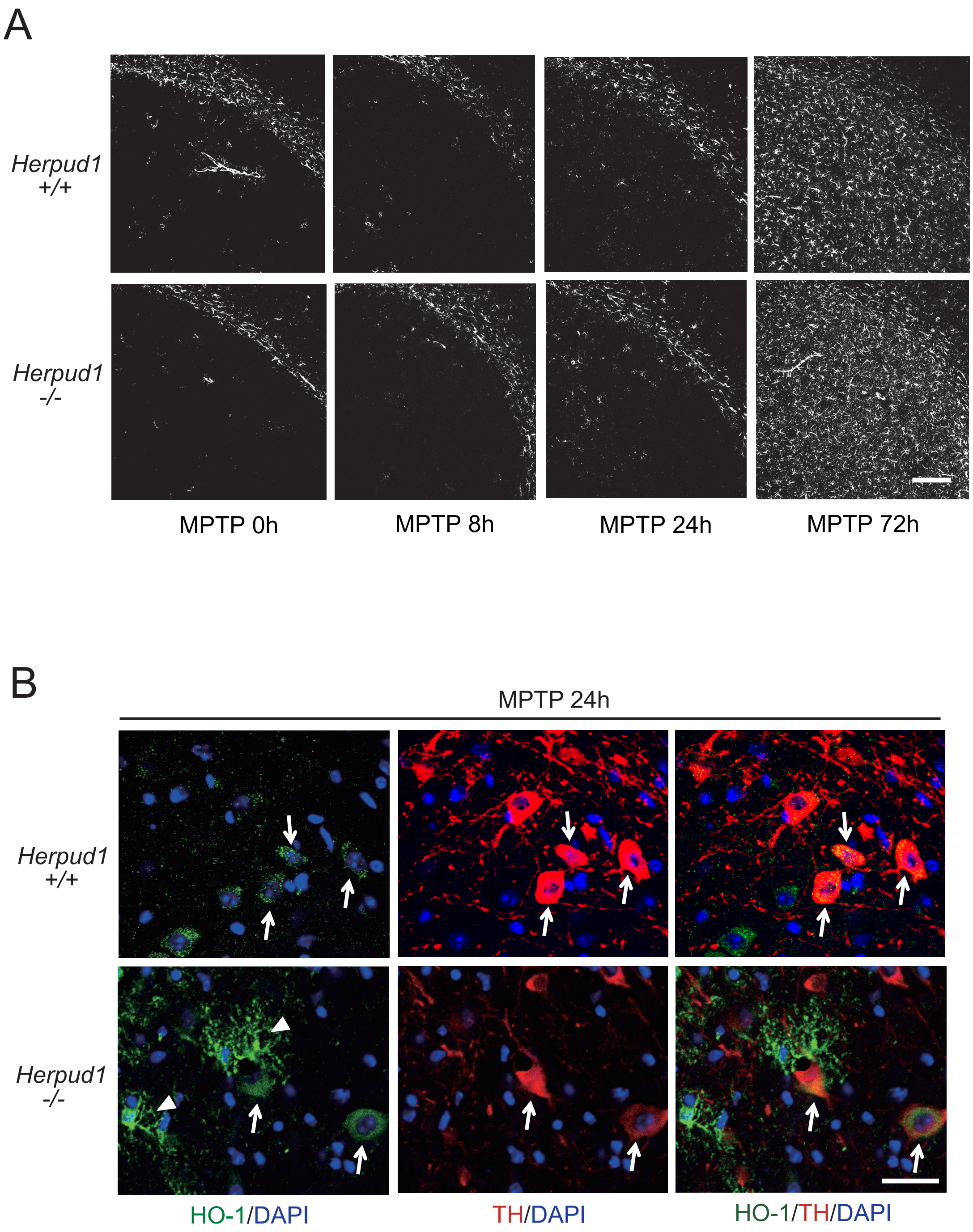
A
Herpud1 +/+
Herpud1 -/-
MPTP 0h
MPTP 8h
MPTP 24h
MPTP 72h
B
MPTP 24h
Herpud1 +/+
Herpud1 -/-
HO-1/DAPI
TH/DAPI
HO-1/TH/DAPI

Fig.S1

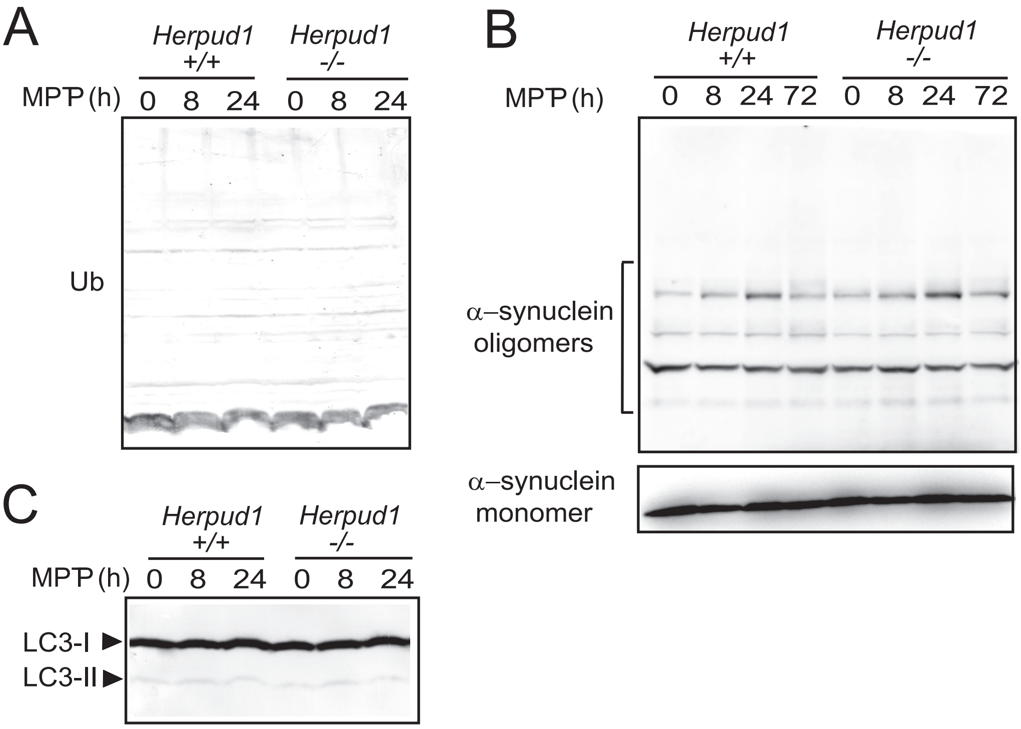

A
Herpud1 +/+
Herpud1 -/-
MPP⁺(h) 0 8 24 0 8 24
Ub
B
Herpud1 +/+
Herpud1 -/-
MPP⁺(h) 0 8 24 72 0 8 24 72
α–synuclein oligomers
α–synuclein monomer
C
Herpud1 +/+
Herpud1 -/-
MPP⁺(h) 0 8 24 0 8 24
LC3-I
LC3-II

Fig.S2

**Potein**	**Gene**	**Forward**	**Reverse**
Herp	*Herpud1*	AGAACATCTCtAGGCCTGAG	TGCCTTGCATAGATCTGCTG
HO-1	*Hmox1*	AGGAGCTGCACCGAAGGGCTG	TCATCTCCAGAGTGTTCATGC
Nrf2	*Nfe2l2*	GATCCGCCAGCTACTCCCAGGTTG	CAGGGCAAGCGACTCATGGTCATC
GRP78	*Hspa5*	ATGGTATTCTCCGAGTGACA	ATGGTATTCTCCGAGTGACA
β-actin	*Actb*	TGTGATGGTGGGAATGGGTCAGAA	TGTGGTGCCAGATCTTCTCCATGT

Supplemental Table1
